# Supplementary material for: Alternative splicing landscapes in Arabidopsis thaliana across tissues and stress conditions highlight major functional differences with animals
Source: Genome Biol. 2021 Jan 14;22:35. doi: 10.1186/s13059-020-02258-y (PMC7807721; doi:10.1186/s13059-020-02258-y)
Supplement: Supplementary file 13 — Additional file 13: Review history. [file 13059_2020_2258_MOESM13_ESM.docx]

Review History

**First round of review**

**Reviewer 1**

**Are you able to assess all statistics in the manuscript, including the appropriateness of statistical tests used?**

Yes, and I have assessed the statistics in my report.

**Comments to author:**

In this manuscript Martin et al. generated the largest transcriptomic dataset to date of Arabidopsis, composed of 522 RNA-seq datasets obtained from different tissues, single cells, and under different growth and stress conditions. The authors generated a web tool available to the scientific community to allow analysis of genes of interest. In addition, the authors examined the effect of stress and different growth conditions on transcription levels and alternative splicing. The major finding is that under certain growth conditions the importance of exon skipping is profound. This is an unexpected result as in plants intron retention is the major alternative splicing event. Overall, this is an important manuscript that should be published in Genome Biology pending some revisions.

Figure 2 is the central piece of this manuscript. It shows the fraction of each alternative splicing event in the different conditions. The major conclusion that emerged from this figure is that exon skipping is a much more important alternative splicing event than intron retention, which was what was anticipated based on data generated so far in Arabidopsis. Thus, this referee expected to see from Figure 3 onward comparison of exon skipping to intron retention. Specially, I would like to see if the exon skipping events that are enriched in Figure 2 are functional: For example, How do these events affect open reading frames? Are the same events conserved in other organisms? Although such functional questions were asked, comparisons between the different types of alternative splicing events are missing, and the results are written more like a description of how to use the web tool than a research paper. This should be addressed when the manuscript is revised.

Minor changes

Page 3 line 1: The word “dozens” might be relevant for plants, but if the authors refer to all multicellular organisms the word “dozens” is unjustified for mammals.

Page 4 line 11: The word “despite” appears twice in the same sentence.

**Reviewer 2**

**Are you able to assess all statistics in the manuscript, including the appropriateness of statistical tests used?**

Yes, and I have assessed the statistics in my report.

**Comments to author:**

The authors described a large scale study that characterizes the Arabidopsis alternative splicing landscape across tissues as well as biotic and abiotic stresses. Interesting differences have been identified when compared with animals. As a result, PastDB is created as a resource for checking gene expression and AS quantifications across different tissues and conditions. The website is well constructed and working well.

Here are the detailed comments:

1) cRPKM is used as measurements in the database. If it follow the same calculation of RPKM, the sum of RPKMs will vary from sample to sample. TPM remains a constant across samples, which is a better measurement to compare across sample. I would suggest to add TPM to the database too.

2) re-using public data leverage the great power of big data. However, our experiences that the RNA-seq data generated from different studies varies greatly in quality. The authors have taken measurement to remove samples with low read counts, but many other issues still could affect the quality of the data, e.g. low mapping rates and significant batch effects, etc. A thorough QC have to be in place before utilizing the data.

3) it would be good to add AS statistics, for example, how many AS events being detected and how does it compared to atRTD2, , which is a more comprehensive transcriptome that TAIR or Araport in terms of AS events https://academic.oup.com/nar/article-lookup/doi/10.1093/nar/gkx267.

4) page 15, line 53, "genes regulated by AS and GE under different conditions do not overlap". I don't agree. Many genes are regulated at gene level and AS level, including key clock genes. There are ~800 genes detected that are under the regulation of both GE and AS in Calixto et al (http://www.plantcell.org/content/30/7/1424).

5) page 18, line 29. Specify the reference transcriptome used.

6) page 18, line 31, why a normalization method for microarray is used for cRPKM measurements for RNA-seq data? There are plenty of tools developed for normalization of RNA-seq data

7) Page 20-21, The definition of different core sets with similar concepts are very specific and varies greatly, e.g. sometimes 2 out of 3, sometimes 2 out of 5. It is not clear how the criteria are decided and how does the choice of these parameter affects the results and interpretations.

8) page 22 line 44, human models were used in Matt. Has the model been tested in plants? how applicable it is to Arabidopsis?

9) I would also advice to make the codes used in this analysis to be available on github to improve reproducibility

**Authors Response**

**Point-by-point responses to the reviewers’ comments:**

REVIEWER 1

In this manuscript Martin et al. generated the largest transcriptomic dataset to date of Arabidopsis, composed of 522 RNA-seq datasets obtained from different tissues, single cells, and under different growth and stress conditions. The authors generated a web tool available to the scientific community to allow analysis of genes of interest. In addition, the authors examined the effect of stress and different growth conditions on transcription levels and alternative splicing. The major finding is that under certain growth conditions the importance of exon skipping is profound. This is an unexpected result as in plants intron retention is the major alternative splicing event. Overall, this is an important manuscript that should be published in Genome Biology pending some revisions.

Figure 2 is the central piece of this manuscript. It shows the fraction of each alternative splicing event in the different conditions. The major conclusion that emerged from this figure is that exon skipping is a much more important alternative splicing event than intron retention, which was what was anticipated based on data generated so far in Arabidopsis. Thus, this referee expected to see from Figure 3 onward comparison of exon skipping to intron retention. Specially, I would like to see if the exon skipping events that are enriched in Figure 2 are functional: For example, How do these events affect open reading frames? Are the same events conserved in other organisms? Although such functional questions were asked, comparisons between the different types of alternative splicing events are missing, and the results are written more like a description of how to use the web tool than a research paper. This should be addressed when the manuscript is revised.

*R: We would like to thank the Reviewer for his/her positive assessment of our work and suggestions for improvement, which we have carefully followed.*

*First, we would like to clarify that we did not intend to claim that exon skipping is more important than intron retention or that the absolute contribution of the former is larger than that of the latter. Our data suggest that the *relative* importance of exon skipping, but also of alternative 5′ and 3′ splice site choices, to regulated alternative splicing (AS) patterns might be higher than previously appreciated. We have carefully checked our manuscript to avoid overstating our conclusions, and apologize if this has caused some misunderstandings.*

*Second, as requested, we have now repeated all major analyses splitting the events by AS type. These results show that the functional enrichments that we reported hold for exon skipping as well as for the other AS types. These data are now presented in the new supplementary Figures S11 and S15.*

*Third, we have investigated the evolutionary conservation of each type of AS. This is indeed a complex task for plants, since resources equivalent to those for animals (e.g. chain alignments for liftOver) are not available. We have thus generated chain alignments for four other Brassicaceae species (Arabidopsis lyrata, Arabis alpina, Camelina sativa and Brassica rapa; spanning 26 million years of evolution) and investigated conservation at the genomic level for each AS core set and event type. The levels of conservation are similar or higher (particularly for cassette exons) than other highly alternatively spliced events, and well above the level of lowly included AS events. The results of this analyses are presented in the new Figure S10. We also attempted to generate chain alignments for non-Brassicaceae species (e.g. Solanum, Oryza, etc.) but they yielded no conservation using the liftOver tool.*

*Moreover, we have also performed a direct comparison between the AS core sets in the four studied species (A. thaliana plus the three animals). In line with one of the main conclusions of our study, we found no conservation whatsoever between plants and animals at the AS event level, and only a very low overlap at the orthologous gene level. These results are shown in the new Figure S29.*

Minor changes

Page 3 line 1: The word “dozens” might be relevant for plants, but if the authors refer to all multicellular organisms the word “dozens” is unjustified for mammals.

*R: Thank you for pointing it out. We have now changed this sentence to: "Complex multicellular organisms generate a myriad of highly specialized cell and tissue types during their lifetime"*

Page 4 line 11: The word “despite” appears twice in the same sentence.

*R: Corrected, thank you.*

REVIEWER 2

Reviewer #2: The authors described a large-scale study that characterizes the Arabidopsis alternative splicing landscape across tissues as well as biotic and abiotic stresses. Interesting differences have been identified when compared with animals. As a result, PastDB is created as a resource for checking gene expression and AS quantifications across different tissues and conditions. The website is well constructed and working well.

*R: We thank the reviewer for his/her positive and thorough revision of our MS.*

Here are the detailed comments:

1) cRPKM is used as measurements in the database. If it follow the same calculation of RPKM, the sum of RPKMs will vary from sample to sample. TPM remains a constant across samples, which is a better measurement to compare across sample. I would suggest to add TPM to the database too.

*R: Thank you for the suggestion. We have now introduced an option to generate TPMs in vast-tools. Moreover, since TPMs can be easily worked out from cRPKM values (TPM = 10^6 * cRPKM/sum_all(cRPKMs)), which can be downloaded from PastDB, we have added a note in the FAQ section and in the tooltip of the expression plots indicating how to generate TPMs from any PastDB expression table.*

*As a sanity check, we have also generated the GE core sets using TPMs instead of cRPKMs. We obtained an overlap of 99%, 98% and 90% for the abiotic, biotic and tissue GE core sets, and very similar gene ontology enrichments. This information has now been added to the section "Definition of the core sets of regulated genes at the GE level".*

*Finally, we would like to point out that the normalization we performed, which is essentially a quantile normalization (see point 6), also makes the sum of all cRPKM values per sample to be nearly identical across samples.*

2) re-using public data leverage the great power of big data. However, our experiences that the RNA-seq data generated from different studies varies greatly in quality. The authors have taken measurement to remove samples with low read counts, but many other issues still could affect the quality of the data, e.g. low mapping rates and significant batch effects, etc. A thorough QC have to be in place before utilizing the data.

*R: We fully agree with the Reviewer that this is key point. Since this has also been a major concern for us from the beginning, we had indeed looked at other features in addition to the number of reads. In particular, we had checked the percent of reads that map to the genome and to the reference transcriptome, as well as an estimate of the 3′ sequencing bias of each RNA-seq sample (provided by vast-tools). We discarded samples that performed poorly in any of these metrics and/or that were generated using ribo-depletion, unless we considered the experiment unique and of high biological interest. We had not included these metrics in the original version for simplicity, but we have now included them in Supplementary Table 1 and highlighted in red font the few cases in which these are poor.*

*In addition, for experiments covering stimuli responses or developmental series, we tracked the expression of known molecular markers to confirm the validity of each experiment. Furthermore, to minimize molecular signatures attributable to batch effects, we included in our set of samples as many independent experiments as possible for any given condition, provided they passed the abovementioned QCs. As an example, in the case of heat treatments, we included five different experiments. Finally, as explained in the original version of the manuscript, all samples were clustered and outlier samples with strong batch effects were discarded.*

*We have now added an additional explanation about the QCs in the corresponding Methods section.*

3) it would be good to add AS statistics, for example, how many AS events being detected and how does it compared to atRTD2, , which is a more comprehensive transcriptome that TAIR or Araport in terms of AS events https://academic.oup.com/nar/article-lookup/doi/10.1093/nar/gkx267.

*R: The number of AS events detected across samples were shown in Figure 1, although we realize this was not clear. We have now modified the figure to make it clearer. Also, as requested, we have performed the comparison with atRTD2, using SUPPA2 to extract the different AS events by type. Depending on the AS type, between 72% (alternative 5′ splice site choices) and 85% (exon skipping) of the events annotated in atRTD2 are present in PastDB. Conversely, between 37% (intron retention) and 65% (alternative 3′ splice site choices) of highly alternatively spliced events across the main PastDB dataset are also annotated as alternative in atRTD2. Please note, however, that atRTD2 and PastDB are not directly comparable and that the range and type of samples in each resource is different. These results have now been included in the section "PastDB website, database and data sources".*

4) page 15, line 53, "genes regulated by AS and GE under different conditions do not overlap". I don't agree. Many genes are regulated at gene level and AS level, including key clock genes. There are ~800 genes detected that are under the regulation of both GE and AS in Calixto et al (http://www.plantcell.org/content/30/7/1424).

*R: Thank you for pointing it out. We would like to clarify, however, that the full sentence was: "we showed that *most* genes regulated by AS and GE under different conditions do not overlap". We did not intend to say that there was no overlap, but that the majority of the genes in those two categories do not overlap. We have reworded the sentence to avoid this potential misunderstanding and apologize for the confusion.*

5) page 18, line 29. Specify the reference transcriptome used.

*R: This information has now been added. We have used the representative annotated transcript per gene from Ensembl Plants v31. The list of transcripts is now provided as Additional file 12: Table S11.*

6) page 18, line 31, why a normalization method for microarray is used for cRPKM measurements for RNA-seq data? There are plenty of tools developed for normalization of RNA-seq data.

*R: We would like to clarify that, although the R package used to perform the normalization is called "normalizeBetweenArrays", and it was indeed originally designed for microarray data, we have used it to do a standard quantile normalization of our data (for example, voom also uses this package). The reason why we have used this normalization method is because it is a fairly simple way to make cRPKMs more comparable across samples (as mentioned in point 1), as it has been done in other RNA-seq-based publications before. We have reworded the corresponding Methods section to clarify the use of normalizeBetweenArrays in the context of a quantile normalization.*

7) Page 20-21, The definition of different core sets with similar concepts are very specific and varies greatly, e.g. sometimes 2 out of 3, sometimes 2 out of 5. It is not clear how the criteria are decided and how does the choice of these parameter affects the results and interpretations.

*R: Thank you for pointing this out. We believe the Reviewer is referring to the difference between the definition of the stress cores and the tissues cores. In this case, the same criteria cannot be applied to both types of cores, since the comparisons are essentially different. For stress conditions, we need to compare a stress condition with its corresponding control. We therefore required an event to change in the same direction in 2 out of 5 experiments. Both abiotic and biotic stress cores were done following the same criteria. For tissues, we do not have this paired design, and each tissue is compared with all others (at least 3). However, to ensure a good representativity of patterns per tissue, we required that, for each compared event, each valid tissue had at least 2 replicates with sufficient read coverage. We agree with the reviewer that this looks a bit complex, so we have now added an additional clarification in the corresponding Methods section.*

8) page 22 line 44, human models were used in Matt. Has the model been tested in plants? how applicable it is to Arabidopsis?

*R: We have used the human model for all species for simplicity, as it had been done before for A. thaliana (e.g. Godoy Herz et al, Mol Cell 2019), and keeping in mind that the consensus sequences are quite comparable between animal and plants. However, the Reviewer is right that this needs to be properly assessed. We have therefore employed a complementary approach and calculated the splice site strengths as the similarity to the position-weighted matrices (PWM) built from all annotated 5' and 3' splice sites from A. thaliana. We have repeated the corresponding analyses with these metrics, obtaining very similar results (presented in the new Figure S32).*

9) I would also advice to make the codes used in this analysis to be available on github to improve reproducibility.

*R: Thank you for the suggestion. Although some of the analyses and plots were computationally simple and produced in Excel, we have created a github repository (https://github.com/vastdb-pastdb/pastdb) and added the code used for the main analyses, as explained in the README file. Furthermore, we provide in this README the download links to all the data hosted in PastDB.*

**Second round of review**

**Reviewer 2**

All the issues raised have been appropriately addressed.
